# Whole-genome sequence of *Haemophilus parainfluenzae* EL1 isolated from healthy supragingival plaque

**DOI:** 10.1128/MRA.00476-23

**Published:** 2023-09-29

**Authors:** Thais H. de Palma, Emily Loomis, Dasith Perera, Matthew Ramsey

**Affiliations:** 1 Department of Cell and Molecular Biology, University of Rhode Island, Kingston, Rhode Island, USA; Wellesley College Department of Biological Sciences, Wellesley, Massachusetts, USA

**Keywords:** oral microbiology, commensal, dental plaque

## Abstract

*Haemophilus parainfluenzae* is a Gram-negative bacterium that colonizes the mouth and the upper respiratory tract. Here, we report the genome sequence of *H. parainfluenzae* strain EL1 isolated from healthy supragingival plaque. This strain is used as a representative commensal of the oral microbiota.

## ANNOUNCEMENT

*Haemophilus parainfluenzae* is a Gram-negative facultative anaerobic bacterium that colonizes different niches in the mouth (1–4) and the upper respiratory tract (1, 5). *H. parainfluenzae* is also an opportunistic pathogen (6–10).

Here, we report the genome sequence of *H. parainfluenzae* strain EL1 isolated from the supragingival plaque of a healthy individual in Rhode Island, USA. This strain was chosen as a representative oral commensal. EL1 collection was approved by the IRB of the University of Rhode Island with informed volunteer consent.

Supragingival plaque was collected from teeth via sterile toothpick and plated on brain heart agar supplemented with 5% yeast extract, 15 mg/mL hemin, 15 mg/mL NAD (BHIYE-HP), and 5 mg/mL vancomycin. Plates were incubated overnight at 37°C and 5% CO_2_. Single colonies were inoculated in 5 mL of BHIYE-HP and incubated overnight. DNA was extracted using DNeasy Blood and Tissue Kit (Qiagen) following manufacturer’s instructions. Quantity and quality of DNA were assessed by Nanodrop. 16S rDNA was amplified using 27F/1492R primers and amplicons Sanger sequenced. Sequences were aligned using BLASTN against the rRNA/ITS NCBI database revealing 99% identity to *H. parainfluenzae* ATCC33392.

The genome was sequenced via Illumina and Oxford Nanopore. Illumina libraries were prepared using the Illumina DNA Prep Kit and samples were indexed using Integrated DNA Technologies (IDT) 10 bp Unique Dual Indexes (UDI). Sequencing was performed using an Illumina NextSeq 2000 producing 2 × 151 bp reads considering quality score ≥Q30. Data were demultiplexed and adapters removed with bcl2fastq v2.20.0.445 (11) (default parameter). The assembly algorithm considers per-base quality scores, assigning weighted scores to components determining which contigs are included in the final assembly. The “normal” conservation threshold was used under default parameters. The total number of paired-end reads was 3,998,695 corresponding to 7,997,390 Illumina reads and 555× coverage. For Nanopore, EL1 was prepared using Oxford Nanopore’s Genomic DNA Ligation Kit (SQK-LSK109) following manufacturer’s instructions and without DNA shearing. Sequencing was done using Nanopore R9 flow cells (R9.4.1) on a MinION. Long-read buffer was used during the magnetic bead wash, selecting fragments >3 kb. Base calling was done by Guppy 4.2.2 with default parameters + effbaf8 using the high accuracy algorithm. There were 32,943 raw Nanopore reads (N50 = 19,919). Adapters, barcodes, and chimeric reads were removed with Porechop v0.2.3_seqan2.1.1 (12) using default parameters. Assembly was done with Unicycler v0.4.8 (13) using “hybrid assembly.” Nanopore reads not aligning to Illumina-produced contigs were removed using Unicycler defaults. Assembly statistics were recorded with QUAST 5.0.2 (14) under default parameters and annotated by NCBI Prokaryotic Genome Annotation Pipeline (15) software v.6.5.

Assembly resulted in one circular contig with length of 2,153,240 bp. The GC content was 39.41%, and the N50 was 2,153,240. In total, 2,086 genes were identified. Of those, 2,005 are coding sequences and 1,990 protein-coding genes. Moreover, 7, 6, 6 (5S, 16S, and 23S) complete RNAs and 58 tRNAs were identified.

## Data Availability

This whole genome shotgun project has been deposited under BioProject PRJNA954350 (BioSample SAMN34140652) and under DDBJ/ENA/GenBank, accession number CP125087, version CP125087.1. Nanopore and Illumina reads can be accessed under the respective SRA accession numbers SRR24129362 and SRR25463078.
